# Draft Genome Sequence of Lactobacillus plantarum EBKLp545, Isolated from Piglet Feces

**DOI:** 10.1128/MRA.01739-18

**Published:** 2019-04-18

**Authors:** Sukjung Choi, Eun Bae Kim

**Affiliations:** a Laboratory of Microbial Genomics and Big Data, College of Animal Life Sciences, Kangwon National University, Chuncheon, Republic of Korea; b Division of Applied Animal Science, College of Animal Life Sciences, Kangwon National University, Chuncheon, Republic of Korea; c Department of Animal Life Science, College of Animal Life Sciences, Kangwon National University, Chuncheon, Republic of Korea; University of Southern California

## Abstract

Lactobacillus plantarum strain EBKLp545 was isolated from piglet feces in South Korea and sequenced using an Illumina HiSeq system. This draft genome of strain EBKLp545 consists of 3,306,513 bp with 3,049 protein-coding genes in 138 contigs (≥500 bp), 54 noncoding RNA genes, and a 44.3% G+C content.

## ANNOUNCEMENT

Lactobacillus plantarum is a Gram-positive and acid-tolerant bacterium that is a major part of the family of lactic acid bacteria (1, 2). L. plantarum is found in a wide variety of habitats and is a commonly used species as a probiotic for livestock benefits because it can survive in the gastrointestinal tract (GIT) and has excellent long-term fixability (3–5). However, only a limited number of genomes from pig feces are available from the NCBI (6). Since the prohibition of feed antibiotics in South Korea in 2011, Lactobacillus genomes from pig feces have changed (7, 8). Recently, after the prohibition of antibiotics, Lactobacillus salivarius strains have improved cell aggregation by obtaining or removing genes for exopolysaccharides (EPS) and extracellular proteins or by mutating such genes (8). *L. plantarum* EBKLp545 was isolated in June 2016, after the antibiotic ban, so its genome will be very useful for understanding genomic changes in *L. plantarum* strains caused by the prohibition of antibiotics in feed.

To obtain pure cultured cells on MRS agar plates, a single colony of *L. plantarum* EBKLp545 was inoculated into MRS broth and incubated at 37°C for 24 h. The genomic DNA was extracted from the pure cultured cells via the G-spin total DNA extraction kit (Intron Biotechnology, South Korea) according to the manufacturer’s instructions. An Illumina sequencing library was constructed with ∼350-bp inserts via the Nextera XT DNA library preparation kit (Illumina, CA), according to the manufacturer’s recommendations. The library was sequenced on the HiSeq 2500 platform (Illumina) to obtain 100-bp paired-end reads. For sequence filtering, we trimmed adapter sequences with Cutadapt v1.14 (9) and selected only the reads in which 95% of bases showed a quality score of ≥31 by using an in-house Perl script. After filtering, the selected 1,227,229 reads (length of ≥70), representing 38-fold coverage of the genome, were assembled using SPAdes v3.9 (10) with default parameters.

**FIG 1:**
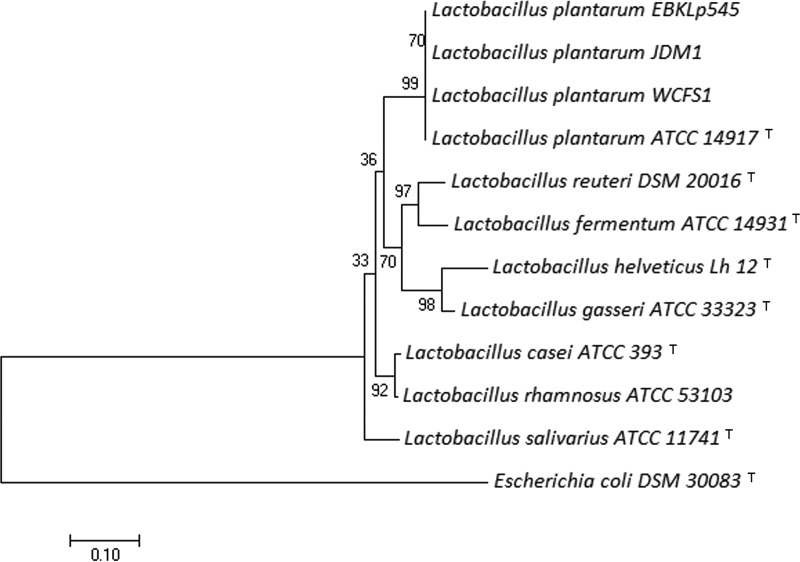
16S rRNA gene phylogenetic tree indicating the position of *L. plantarum* EBKLp545 relative to that of other *Lactobacillus* species. Escherichia coli was included as an outlier. The optimal tree with a sum of branch length of 1.67 is shown. The percentages of replicate trees in which taxa clustered together in the bootstrap test (1,000 replicates) are shown next to the branches. ^T^, type strain.

The draft genome consisted of 3,306,513 bp in 138 contigs (of more than 500 bp), with an *N*_50_ value of 61,792 bp, and a G+C content of 44.3%, all of which were similar to those of other *L. plantarum* genomes uploaded to the NCBI genome database (6). The assembled genome was deposited in the NCBI database. Annotation was automatically conducted and added to the genome by the NCBI Prokaryotic Genome Annotation Pipeline (PGAP) (11). A total of 3,246 genes were identified, which included 3,049 protein coding sequences (CDS), 54 noncoding RNA genes (48 tRNA and 3 rRNA), and 143 pseudogenes. For genome comparison, we used Rapid Annotation of microbial genomes using Subsystems Technology (RAST) (12)-annotated genomes, and we found that our draft genome has a different gene composition than that of the 10 other existing *L*. *plantarum* strains in the NCBI database (WCFS1, NC_004567; ZS2058, CP012343; ST_III, NC_014554; P_8, NC_021224; LZ95, CP012122; JDM1, NC_012984; JBE245, CP014780; DOMLa, CP004406; 5_2, CP009236; and 16, CP006033). Although genes related to programmed cell death and the toxin-antitoxin system were abundant, protein biosynthesis (tRNA) and carbohydrate (l-arabinose, l-rhamnose, and fructooligosaccharide) utilization genes were found to be deficient compared to other strains. We also screened the genome to assess whether there were any known antibiotic resistance genes collected by the Antibiotic Resistance Genes Database (ARDB) (13). Only one antibiotic resistance gene (*bacA*) was found, and it was responsible for resistance against bacitracin; it was also found in 10 other *L. plantarum* strains. To investigate the evolutionary relationships with other *Lactobacillus* species, a phylogenetic tree was constructed using 16S rRNA gene sequences (Fig. 1). 16S rRNA gene sequences of eight *Lactobacillus* species were aligned using ClustalW with default settings, and the phylogenetic tree was constructed using Molecular Evolutionary Genetics Analysis v7 (MEGA7) (14) by the neighbor-joining method. As expected, *L. plantarum* EBKLp545 was classified as a strain of *L. plantarum*. The average nucleotide identity based on BLAST+ (ANIb) was calculated by JSpeciesWS v3.0.20 (15). The comparison to the *L. plantarum* ATCC 14917 type strain showed over 98% ANIb. More piglet strains are required to be genome sequenced to determine whether these characteristics are unique features of *L. plantarum* bacteria derived from piglet feces.

### Data availability.

The draft genome sequence for the strain obtained from pig feces has been deposited in GenBank under BioProject number PRJNA448371, BioSample number SAMN08828920, and accession number PZPN00000000. Sequence data have been deposited in the Sequence Read Archive under the accession number SRP168492.
